# Dose perturbation effect of metallic spinal implants in proton beam therapy

**DOI:** 10.1120/jacmp.v16i5.5566

**Published:** 2015-09-08

**Authors:** Yingcui Jia, Li Zhao, Chee‐Wai Cheng, Mark W. McDonald, Indra J. Das

**Affiliations:** ^1^ Radiation Oncology Indiana University School of Medicine Indianapolis IN; ^2^ ProCure Proton Therapy Center Oklahoma City OK; ^3^ University Hospital/Case Medical Center Cleveland OH; ^4^ Indiana University Health Proton Therapy Center Bloomington IN USA

**Keywords:** dose perturbation, metallic spinal implants, proton therapy

## Abstract

The purpose of this study was to investigate the effect of dose perturbations for two metallic spinal screw implants in proton beam therapy in the perpendicular and parallel beam geometry. A 5.5 mm (diameter) by 45 mm (length) stainless steel (SS) screw and a 5.5 mm by 35 mm titanium (Ti) screw commonly used for spinal fixation were CT‐scanned in a hybrid phantom of water and solid water. The CT data were processed with an orthopedic metal artifact reduction (O‐MAR) algorithm. Treatment plans were generated for each metal screw with a proton beam oriented, first parallel and then perpendicular, to the longitudinal axis of the screw. The calculated dose profiles were compared with measured results from a plane‐parallel ion chamber and Gafchromic EBT2 films. For the perpendicular setup, the measured dose immediately downstream from the screw exhibited dose enhancement up to 12% for SS and 8% for Ti, respectively, but such dose perturbation was not observed outside the lateral edges of the screws. The TPS showed 5% and 2% dose reductions immediately at the interface for the SS and Ti screws, respectively, and up to 9% dose enhancements within 1 cm outside of the lateral edges of the screws. The measured dose enhancement was only observed within 5 mm from the interface along the beam path. At deeper depths, the lateral dose profiles appeared to be similar between the measurement and TPS, with dose reduction in the screw shadow region and dose enhancement within 1–2 cm outside of the lateral edges of the metals. For the parallel setup, no significant dose perturbation was detected at lateral distance beyond 3 mm away from both screws. Significant dose discrepancies exist between TPS calculations and ion chamber and film measurements in close proximity of high‐Z inhomogeneities. The observed dose enhancement effect with proton therapy is not correctly modeled by TPS. An extra measure of caution should be taken when evaluating dosimetry with spinal metallic implants.

PACS number: 87.50.sj

## I. INTRODUCTION

Proton beam therapy with 50–250 MeV is widely used in the treatment of brain tumors, skull‐based chordoma, spine chordoma, prostate adenocarcinoma, medulloblastoma, retinoblastoma, and lung cancers. The rationale for proton treatment is to deliver high dose to the target and spare adjacent healthy tissues. With the increased utilization of proton therapy, there will be more patients receiving proton radiation therapy with high‐Z implants such as dental alloys, hip prosthesis, prostate gold markers, and spinal metallic screws.

Modern radiotherapy uses X‐ray computed tomography (CT) for most treatment planning. The presence of high‐Z materials causes severe artifacts in the CT images, masking the true values of CT numbers at the affected pixels and, more importantly, rendering accurate delineation of the high‐Z heterogeneity a very challenging task. Indeed, it has been reported that knowing the size and shape of a high‐Z heterogeneity is more important than the accuracy of knowing the electron density and, hence, the relative stopping power.^(1)^ The problem is enhanced in proton therapy if a metal artifact‐contaminated CT dataset is used for dose calculations. The presence of high‐Z materials in the proton beam causes perturbations in the dose distribution. Several groups have studied the dose perturbation in proton radiotherapy from various fiducial markers such as gold, stainless steel, and titanium.^2^, ^3^, ^4^, ^5^, ^6^, ^7^ They demonstrated that metal fiducials cause dose perturbation in dose distribution and that the dose perturbation in proton therapy is dependent on marker size, placement depth, and orientation with respect to proton beam axis. Newhauser et al.^(2)^ recommended a 0.9 mm diameter, 3.1 mm long cylindrical stainless steel marker to serve as a prostate fiducial marker in proton beam treatment. Examples of potential severe proton beam range errors and target underdosage in the presence of metal have also been reported.^8^, ^9^

To reduce the proton range uncertainties in treatment plans for patients with high‐Z implants, a hybrid planning approach using MVCT and kVCT has been reported.^(10)^ On the other hand, a more fundamental approach aiming to reduce the metallic artifacts in CT scans was proposed as early as 1981.^(11)^ Various metal artifact reduction algorithms have since been published.^12^, ^13^, ^14^ Metal artifact suppression algorithms in CT studies for proton therapy have also been reported.^9^, ^15^

The magnitude of dose perturbation from spinal metallic implants is greater than those caused by fiducial markers because of the significantly larger size of spine stabilization hardware and similar implants. Analyses of clinical outcomes of postoperative proton therapy for extracranial chordoma and spinal sarcomas found that the presence of spinal stabilization hardware and metallic implants was associated with an increased rate of local failure, and complicated the delivery of proton therapy.^16^, ^17^, ^18^ To the best of our knowledge, there is no published data on the magnitude of the dose perturbation from a spinal metallic implant in proton therapy.

The objective of this study was to investigate the effects of the dose perturbations in proton beam therapy from two commonly used spinal metallic screw implants using experimental measurements and compare them with calculations using a proton treatment planning system. The metal artifact reduction effect on the dose distribution in the treatment planning system was also investigated.

## II. MATERIALS AND METHODS

### A. Experimental setup and measurement

**Figure 1:**
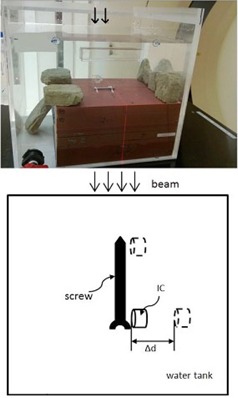
Experimental setup for the CT scan and dose measurement. Top: A proton beam was incident perpendicular to the metallic screw (the arrows on top of the phantom indicate the proton beam incident direction at the perpendicular setup). Several Gafchromic films were placed at various depths (at several depths (0–73 mm) between different solid water slabs. Bottom: A proton beam was incident parallel to the screw longitudinal axis. A plane‐parallel ion chamber (IC) was used for the measurement along the transverse axis as delta distance increases. This measurement was repeated at the tip and also at just above the arms of the screw.

Two commonly used fixation screws — a stainless steel (SS) screw (CD horizon spinal system, fixed angle bone screw, size 5.5 mm×45 mm, M8, MAT'L: ASTM 138 stainless, REF 858‐545, LOT 87633; Medtronic Sofamor Danek, Memphis, TN) and a titanium (Ti) screw (CD horizon spinal system, fixed angle screw, size: 5.5 mm×35 mm, MAT'L: ASTM F136 Ti‐6Al‐4V, REF 7543535, LOT H07B0873; Medtronic Sofamor Danek) — were used in this study. Figure 1 shows the experimental setup for both the CT scan and dose measurement. A proton beam was incident perpendicular to the metallic screw, as shown on the top of the phantom. Several Gafchromic EBT2 films (International Specialty Products, Wayne, NJ) were placed at various depths (0–73 mm) in the Solid Water phantom to measure the dose distributions in the distal planes. The cross‐sectional area of the solid water slabs is 30 cm×30 cm. The CT scanned area was chosen large enough to accommodate the screws and small enough to remove the effects from rocks which were used to stabilize the solid waters in the water phantom. A second experimental setup for CT scan was with the screw's longitudinal axis perpendicular to the solid water surface. For dose measurement, this second setup was adopted in a large water tank, with proton beam incidence along the screw longitudinal axis (see the lower image of Fig. 1). A plane‐parallel ion chamber (Advanced Markus chamber, serial number 1031, active volume 0.02 cm^3;^ PTW, Freiburg, Germany) was used for the measurement. The ion chamber was placed as close as possible to the metal screw without collision. That location was described as delta distance zero. The ion chamber was moved transversely to different locations along the lateral profile as the delta distance increases. This measurement was repeated both at the tip end and at just above the arms of the screw.

**Figure 2:**
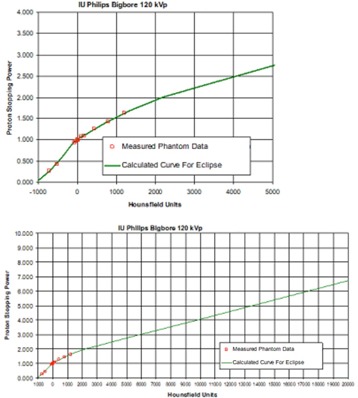
IU Philips Bigbore CT scanner calibration curves; (upper) the manufacture default curve used in proton treatment; (lower) the scanner extends the Hounsfield units (HU) to cover those of the high‐Z materials.

A Philips Big Bore CT scanner (Philips Healthcare, Andover, MA) at the Department of Radiation Oncology, Indiana University was used to acquire CT scans of each geometry in 1 mm slice thickness, using an orthopedic metal artifact reduction algorithm (O‐MAR). A detailed description of O‐MAR is described in a white paper from Philips Healthcare.^(19)^ The CT scanner default Hounsfield unit (HU) range is from −1000 to 5000. In order to cover the high‐Z materials, the HU was extended to 20000 (extended scale), as shown in Fig. 2. A HU of 16000 was assigned to the SS to give a proton relative linear stopping power of 5.66. A HU of 6700 was assigned to the Ti screw to give a proton relative linear stopping power of 3.20. In this study, the relative stopping power for protons in various materials (steel, titanium, and water) were calculated using the Bethe‐Bloch equation and compared with the corresponding NIST data.

### B. Metal contouring and treatment planning

A treatment plan was generated using the Varian Eclipse treatment planning system (TPS) (version 10.0.25; Varian Medical Systems, Palo Alto, CA) at the Indiana University Health Proton Therapy Center in Bloomington, Indiana, for each setup geometry. The TPS‐planned lateral and distal dose profiles were compared with measurements using an Advanced Markus plane‐parallel ion chamber, as well as the film dosimetry data from the Gafchromic EBT2 films.

The CT dataset with O‐MAR provided the baseline data for the metallic screws (diameter and HU). This information is used to contour the screw in the Eclipse TPS for the CT dataset without MAR. The plans, with and without O‐MAR, had the same isocenter and the dose was normalized to the same point. The known artifact around the metal was contoured and assigned to the proton stopping power of water. A proximal margin of 2 cm and a distal margin of 9 cm from the isocenter were chosen for the plans. A calculation grid size of 1 mm×1 mm×1 mm was applied in the TPS for better dose space resolution (the default is 2.5 mm). The standard condition at the Indiana University Health Proton Therapy Center gantry rooms was used for the measurement and planning. The standard condition is defined as 16 cm beam range in water and 10 cm spread‐out Bragg Peak (SOBP) using the 10 cm snout and a $12\times 12\;{\text{cm}}^{2}$ scanning pattern. The ion chamber or any detector is placed at 11 cm depth on the central axis of the beam, which corresponds to the center of the SOBP. An air gap of 5 cm is maintained between the aperture and the water phantom front face. A compensator was not used for the measurements or the treatment planning.

### C. Gafchromic EBT2 dosimetry

Gafchromic EBT2 films, which were used to study the dose profile in the distal region of the dose distribution in the perpendicular setup, were calibrated under the standard condition. Due to batch‐to‐batch variability in Gafchromic film, a calibration curve was created for each batch of EBT2 films using the same beam irradiation conditions. The films in our measurement were all located within the SOBP plateau, from d=0 mm (directly underneath the screw edge) to d=73 mm.

**Figure 3:**
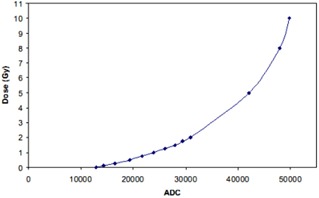
Gafchromic EBT2 film calibration curve in OmniPro.

Twenty‐four hours after irradiation, the irradiated films were scanned in a photographic scanner (Epson Expression 10000XL; Epson America, Long beach, CA) using a setup of positive film type, 48‐bit RGB color image, and 150 dpi resolution. The red‐channel of the scanned images was extracted by ImageJ software and then imported into the OmniPro I'MRT (SW version 1.4b; Scanditronix Wellhofer, Schuarzenbruck, Germany) for analysis. Figure 3 shows the calibration curve for the Gafchromic EBT2 films, a plot of the known doses (in gray) versus the signal analog‐to‐digital convertor (ADC) from OmniPro. The measured dose of the films in the experiment was derived based on the ADC signals and the calibration curve. In the final results, the relative dose was used in the comparison.

## III. RESULTS

**Figure 4:**
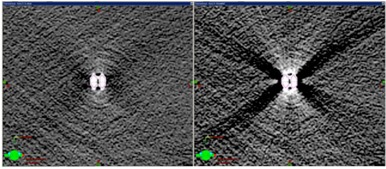
MAR effect on the same CT slice of the stainless steel screw cross section. Left image was processed with MAR and right image was the original.

**Figure 5:**
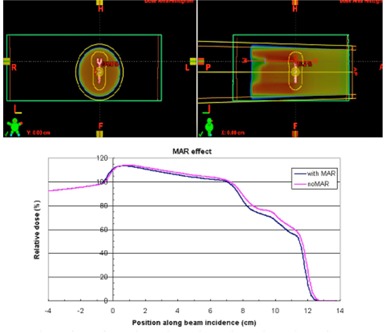
MAR effect on the treatment plans. The distal dose distribution in blue color was from the treatment plan using MAR reconstructed CT data; the distal dose distribution in pink color was from the treatment plan using the original CT set without process of MAR.

Metal artifact reduction software has been employed to improve CT image quality when the metal‐introduced image artifacts are presented. O‐MAR substantially reduces the streaking artifacts surrounding the spinal metallic implants, as shown in Fig. 4. The difference between the dose distributions from the original CT set and the reconstructed CT set by O‐MAR is dependent on the contouring of the metals. Figure 5 shows the relative depth dose distributions for the treatment plans using stainless steel screw CT data processed with and without O‐MAR. The discrepancies between the plans were 2 mm at 20% relative dose and 4 mm at 80% relative dose.

6, 7 show the dose profiles immediately and at a depth of 22 mm downstream from the edge of the stainless steel screw, respectively, in the setup of the beam incidence perpendicular to the longitudinal axis of the screw. Figure 8 is the dose profile comparison between measurement and TPS immediately downstream from the edge of the titanium screw in the perpendicular setup. The measured dose immediately downstream from the screw exhibited dose enhancement up to 12% and 8% for SS and Ti, respectively, but dose perturbation was not observed outside of the edges of the screws. The TPS showed 5% and 2% dose reductions immediately underneath the SS and Ti screws, respectively, and up to 9% dose enhancements within 1 cm outside of the edges of the screws. The measured dose enhancement was only observed within 5 mm from the downstream edge of the screw. At deeper depths, the lateral dose profiles appeared to have similar patterns between the measurement and TPS, with dose reduction in the screw shadow region and dose enhancement within 1–2 cm outside of the edges of the metals.

For the parallel setup, no significant dose perturbation is detected at points over 3 mm laterally beyond the edge of both SS and Ti screws, as shown in 9, 10. Considering the error bars in the data, we cannot distinguish any difference from the background (noise), regardless of whether the measurement was made just above the arms of the screws or at the tip ends of the screws. The TPS data for SS and Ti screws were also plotted in the 9, 10; however, we cannot make any comparison due to the nondetectable dose perturbation using the Advanced Markus plane‐parallel ion chamber.

**Figure 6:**
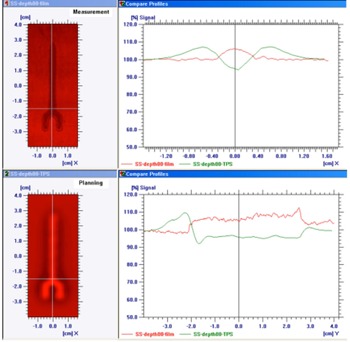
The dose profiles immediately downstream from the edge of the steel screw in the setup of the beam incidence perpendicular to the longitudinal axis of the screw are shown. The upper‐left picture was from the film measurement; the lower‐left picture was from the Eclipse treatment planning system (TPS). The dose profiles along the x‐axis were plotted in the upper‐right frame; the dose profiles along y‐axis (along the longitudinal screw axis) were in the lower‐right frame. Measurement showed dose enhancement immediately downstream from the screw and no dose perturbation outside of the shadow of the screws; TPS showed dose reductions immediately underneath the screws and dose enhancements within 1cm outside of the shadow of the screw.

**Figure 7:**
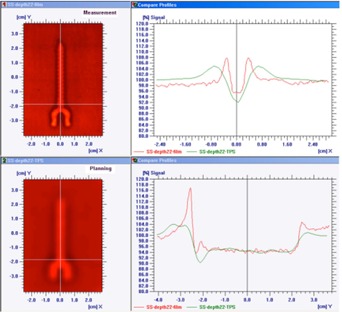
The dose profiles at the depth of 22 mm downstream from the edge of the steel screw in the setup of the beam incidence perpendicular to the longitudinal axis of the screw. The upper‐left picture was from the film measurement; the lower‐left picture was from the TPS. The dose profiles along the x‐axis were plotted in the upper‐right frame; the dose profiles along y‐axis were in the lower‐right frame.

**Figure 8:**
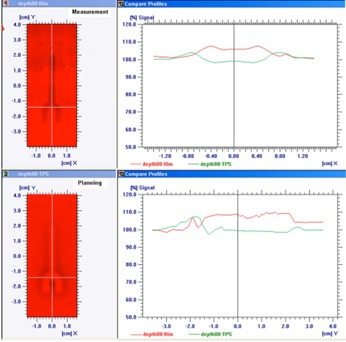
The dose profiles immediately downstream from the edge of the titanium screw in the setup of the beam incidence perpendicular to the longitudinal axis of the screw. The upper‐left picture was from the film measurement; the lower‐left picture was from the Eclipse TPS. The dose profiles along the x‐axis were plotted in the upper‐right frame; the dose profiles along y‐axis (along the longitudinal screw axis) were in the lower‐right frame. Measurement showed dose enhancement immediately downstream from the screw; TPS showed dose reductions immediately underneath the screws and dose enhancements within 1cm outside of the shadow of the screw.

**Figure 9:**
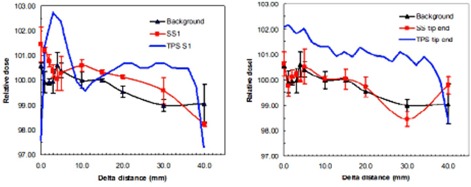
No significant dose perturbation is detected at points over 3 mm laterally beyond the edge of SS screw in the parallel setup, regardless whether the measurement was made at just above the arms (left figure) or at the tip end of SS screw (right figure). SS1 means that dose measurement was made at just above the arms of the stainless steel screw.

**Figure 10:**
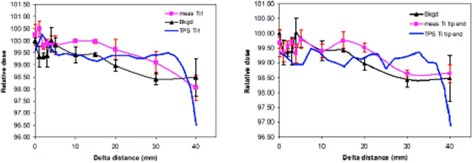
No significant dose perturbation is detected at points over 3 mm laterally beyond the edge of Ti screw in the parallel setup, regardless whether the measurement was made at just above the arms (left figure) or at the tip end of Ti screw (right figure). Ti1 means that dose measurement was made at just above the titanium screw arms.

## IV. DISCUSSION & CONCLUSION

Streaking artifacts in the planning CT images were significantly suppressed with O‐MAR, which is consistent with published reports.^15^, ^19^ Besides Philips, many vendors have included metal artifact reduction in their CT software, such as the Smart Metal Artifact Reduction (SMART) provided by GE, the Metal Deletion Technique (MDT) provided by ReVision Radiology, and the iterative metal artifact reduction algorithm on Siemens platforms. With different algorithms used in different metal artifact reduction methods, user independent verification with known phantom geometry is very important, as the artifact reduced image usually works on small implants but typically results in lower image quality due to the decreased resolution for large or long implants. The ground truth for this study was the known geometry of the phantom setup. This allowed us to correct the image artifact in the water phantom.

Newhauser et al.^(10)^ has studied using MVCT‐kVCT hybrid approach to improve the accuracy of proton range calculation involving the metallic implant. The primary focus in this study was the dose perturbation effect at the metal–tissue interface which has not been studied before. Our approach of handling the range shift of the metallic screws in this study was that the appropriate proton stopping power values are assigned to each screw in the treatment planning system. Since we know the exact dimension of the screws, it allows us to calculate proper therapeutic proton range shift in the treatment planning system. This method has been reported in our previous study.^(20)^

In general, dose distribution can be measured by means of ion chambers, solid state detectors or radiographic films. Of these detectors, the ion chamber is the most reliable in a water phantom mainly because of its relatively flat energy response and precision. In our measurement we did not detect the obvious dose perturbation in the parallel setup, presumably, at least in part, because the plane‐parallel ion chamber we used has a relatively large active volume which would potentially average out dose perturbation effect in the measurements. The ion chamber waterproof cap also limited the closest distance that can be used for the measurement. In order to capture the dose perturbation right at the inhomogeneity interface, the detector shall introduce minimal perturbation on the dose distribution itself with a small volume. Gafchromic film was selected as the dosimeter to study the difference between measurement and treatment planning system for lateral dose profiles due to its high spatial resolution and weak energy dependence. For future improvement, new 3D dosimeters, such as gel dosimeter, could be potentially used for improved spatial resolution with metallic screw imbedded in the gel dosimeter.

Significant dose discrepancies in the close proximity of the high‐Z inhomogeneities in the perpendicular setup between measurements and the TPS are clearly visible even with the O‐MAR CT scans. At the metal–water interface, the dose enhancement in our measurement is probably due to the secondary electron fluence perturbation.^(7)^ This effect is not accurately modeled in the pencil beam algorithm treatment planning system. Clinically, it could cause unwanted excess dose if a critical structure, such as the spinal cord, is right underneath a metallic screw or other hardware. Limitations in the treatment planning system are the main sources of error. In treatment planning, the margin to spare the organ at risk (OAR) is usually 2 to 10 mm. In treatment, the dose perturbation caused by the metallic implants may result in higher dose in the OAR in proton therapy. Our work showed that dose enhancement effect is not correctly modeled by TPS in proton therapy, thus extra caution should be given for evaluating dosimetry in the presence of metallic implants. As Monte Carlo‐based treatment planning systems become more widely available in the future, dose calculation in the presence of high‐Z material at the metal–tissue interface should be more accurately estimated.

An alternative option to reduce the dose perturbation of spinal implants in proton beam therapy is to use nonmetallic implants, such as polyether ether ketone (PEEK) rods and cages, to reduce artifacts in postoperative imaging and tumor surveillance and to improve the accuracy of treatment planning. However, questions regarding the biomechanical stability and long‐term durability of these materials^(21)^ mean they are not suitable for all cases.

## ACKNOWLEDGMENTS

The authors would like to acknowledge Senior Dosimetrist Joe Simmons for the suggestion on the metal contouring and Senior Physicist Chris Allgower for the advice on the treatment planning.
